# Loneliness, Isolation, Cognition, and Mortality Risk: Harmonization Within a Coordinated Analysis Framework

**DOI:** 10.1093/geroni/igaf122.1683

**Published:** 2025-12-31

**Authors:** Tomiko Yoneda, Kathryn Jackson, Katy Bedjeti, Graciela Muniz-Terrera, Daniel Mroczek, Andrew Steptoe, Anthony Ong, Eileen Graham

**Affiliations:** University of California Davis, Davis, California, United States; Northwestern University, Evanston, Illinois, United States; Northwestern University, Evanston, Illinois, United States; Ohio University, Athens, Ohio, United States; Northwestern University, Evanston, Illinois, United States; University College London, London, England, United Kingdom; Cornell University, Ithaca, New York, United States; Northwestern University, Evanston, Illinois, United States

## Abstract

Loneliness and social isolation are associated with several negative health outcomes in older adulthood, including adverse cognitive outcomes and increased mortality risk. Between-study variability in how these constructs are defined and modeled poses a major challenge, potentially contributing to inconsistencies in previous findings. In response, this registered report leveraged a coordinated data analysis framework, applying independent but harmonized operational definitions and statistical models across 11 longitudinal studies of aging (N > 110,000) representing 20 countries. Using Cox regression, logistic regression, and multistate survival models (MSMs), we systematically examined how loneliness and social isolation—both separately and in combination—are associated with cognitive aging outcomes (no cognitive impairment, mild cognitive impairment, severe cognitive impairment) and mortality risk. Preliminary synthesized results based on random-effects meta-analysis show that loneliness and isolation both predict increased risk of dementia and death in Cox and logistic models (all ps < 0.001), with isolation showing a particularly strong link to mortality. In MSMs, however, loneliness (but not isolation) was associated with an increased risk of transitioning from no impairment to mild cognitive impairment. Loneliness and isolation were also associated with an increased risk of transitioning from mild cognitive impairment to death, but this was only true for isolation when loneliness was included in the model. Conversely, isolation appears to be most important for the transition from mild to severe cognitive impairment. Discussion will focus on the evidence for and against the generalizability and variability in associations among social disparities, cognitive outcomes, and mortality risk across individuals, time, and countries.

